# Interest of hepatic steatosis index (HSI) in screening for metabolic steatopathy in patients with type 2 diabetes

**DOI:** 10.11604/pamj.2020.37.270.9087

**Published:** 2020-11-25

**Authors:** Halima Fennoun, Souhaila El Mansouri, Mohammed Tahiri, Nassim Essabah Haraj, Siham El Aziz, Fouad Hadad, Wafaa Hliwa, Wafaa Badr, Asma Chadli

**Affiliations:** 1 Endocrinology, Diabetology and Metabolic Disease Department Ibn Rochd, University Hospital of Casablanca, Casablanca, Morocco,; 2 Hepatology- Gastrology- Enterology Department, Ibn Rochd University Hospital of Casablanca, Casablanca, Morocco

**Keywords:** Metabolic, steatopathy, diabetes, screening, HSI score, transaminases, liver ultrasound

## Abstract

**Introduction:**

metabolic steatopathy or non-alcoholic fatty liver disease (NAFLD) is frequently associated with type 2 diabetes mellitus (T2DM) with an increased risk of progression to advanced fibrosis. The purpose of our study was to determine the interest of hepatic steatosis index (HSI) in the detection of hepatic steatosis in patients with type 2 diabetes in order to establish an appropriate screening program of this disease in our population.

**Methods:**

cross-sectional study involving 281 type 2 diabetics hospitalized in the Department of Endocrinology in collaboration with the Hepato-gastroenterology Department at the University Hospital Ibn Rochd Casablanca between January 2018 and June 2018. Anthropometric variables studied were, biological, hepatic steatosis index (HSI) and liver ultrasound. The HSI score of> 36 predicted the presence of fatty liver. The HSI score (fatty liver index) was calculated for all patients using the following formula: 8 × (ALT / AST) + BMI + 2 (if type 2 diabetes) + 2 (if female). Statistical analysis was performed with SPSS Version 19 software. The sensitivity and the specificity of the HSI score were calculated by 2x2 contingency table. The area under the receiver operating characteristic curve (AUROC) was also analyzed.

**Results:**

average age of patients was 54.15 ± 13.14 years with a female predominance (76.9% of cases), and a sex ratio of 3.32. Mean duration of diabetes of 10.5 ± 8.03 years with an average glycated hemoglobin of 10.23 ± 1.96%. BMI was 29.53 ± 4.55 kg/m^2^, the average waist circumference was 99.51 ± 10.98 cm. 39.1% of patients were hypertensive, 58% were dyslipidemic. Abnormalities in transaminases were found in 6% of patients. Prevalence of NAFLD was 45.2% based on the HSI score > 36. This prevalence is consistent with the findings made by the liver ultrasound (47.7% of cases). Hepatic steatosis was significantly correlated with dyslipidemia (P=0.006), overweight (P=0.00015), obesity (P=0.001) and hypertriglyceridemia (P=0.0003). The sensitivity of HSI was 89.55%, negative predictive value (NPV) was 90.91%, specificity was 95.24%, and positive predictive value (PPV) was 94.49%. AUROC for HSI was at 0.979 (95% CI, 0.962-0.997).

**Conclusion:**

hepatic steatosis is common among our patients; it is correlated with dyslipidemia, obesity and hypertriglyceridemia.

## Introduction

Metabolic steatopathy is a global public health problem; it is the leading cause of chronic hepatopathies in Western countries. It encompasses several disorders ranging from simple hepatic steatosis to nonalcoholic fatty liver hepatitis (NASH), fibrosis and liver cirrhosis. Its association with type 2 diabetes is common [1]. Indeed, it is estimated that about 62%, or two-thirds of type 2 diabetes are hepatic steatosis and 20 to 30% of patients with diabetes are hepatic steatosis [2,3]. Metabolic fatty liver is associated with a high risk of morbidity and mortality related to liver damage severity of imposing early detection to prevent these complications. HSI is a non-invasive marker to identify patients at high risk of fatty liver. The objective of this study is to determine the value of non-invasive score HSI in screening for hepatic steatosis in patients with type 2 diabetes in order to establish an appropriate screening program of this disease in our population.

## Methods

This is a cross-sectional study involving 281 type 2 diabetics hospitalized in the Department of Endocrinology in collaboration with the hepato-gastroenterology department at the University Hospital Ibn Rochd Casablanca between January 2018 and June 2018. The study included any unknown type 2 diabetic patients followed for chronic hepatopathy, or notion of taking hepatotoxic drugs nor alcohol abuse. The variables were studied: age, sex, duration of diabetes, diabetes treatment, glycemic control, cardiovascular risk factors associated with the laboratory tests and liver ultrasound. The HSI score (Hepatic steatosis index) was calculated in all patients using the following formula: 8×(ALT / AST) + BMI + 2 (if type 2 diabetes) + 2 (if female). HSI score of > 36 predicted the presence of hepatic steatosis. Data collection is based on a detailed operating record. For statistical analysis, SPSS version 19 was used. The sensitivity and the specificity of the HSI score were calculated by contingency table. The area under the receiver operating characteristic curve (AUROC) was also analyzed.

## Results

**Figure 1:**
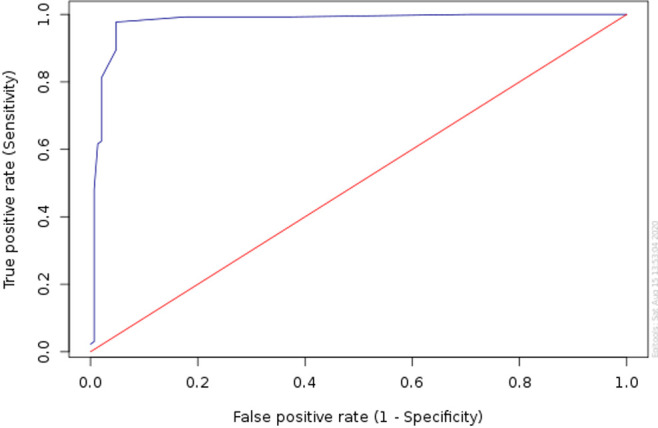
ROC curve for the HSI score

The prevalence of NAFLD was 45.2% based on the HSI score > 36. It is consistent with the findings made by the liver ultrasound (47.7% of cases). The average age of patients was 54.15 ± 13.14 years with a female predominance (76.9% of cases) and a sex ratio of 3.32. The mean duration of diabetes was 10.5 ± 8.03 years with average glycated hemoglobin of 10.23 ± 1.96%. BMI was 29.53 ± 4.55 kg/m^2^, the average waist circumference was 99.51 ± 10.98 cm. 39.1% of patients were hypertensive, 58% were dyslipidemic (Table 1). Abnormalities in transaminases were found in 6% of patients. Hepatic steatosis was significantly correlated with dyslipidemia (P=0.006), overweight (P=0.00015), obesity (P=0.001) and hypertriglyceridemia (P=0.0003). The Table 2 shows risk factors in diabetics with hepatic steatosis on ultrasound compared to those without hepatic steatosis. In multivariate analysis, the factor associated to hepatic steatosis was ALT (P=0.036, odds ratio: 1.143). The sensitivity of HSI was 89.55%, NPV was 90.91%, specificity was 95.24%, and PPV was 94.4% based on data of contingency table (Table 3). The AUROC for HSI was at 0.979 (95% CI, 0.962-0.997) (Figure 1).

**Table 1:** general clinical and biological characteristics of diabetic patients

Characteristics	
Average age (years)	54.15 ± 13.14
Sex ratio	3.32
Women n (%)	216 (76.9)
Men n (%)	65 (23.1)
Mean duration of diabetes (years)	10.5 ± 8.03
Dyslipidemia n (%)	163 (58)
Hypertension, n (%)	110 (39.1)
Mean BMI (kg / m2)	29.53± 4.55
Average waist circumference	99.51± 10.98
Average rate of HbA1c (%)	10.23± 1.96
- ALAT (IU / l)	20.42 ± 17.60
- ASAT (IU / l)	24.41 ± 19.76

**Table 2:** risk factors in diabetics with hepatic steatosis on ultrasound compared to those without hepatic steatosis

	Diabetics with hepatic steatosis N: 134 (47.7%)	Diabetics without hepatic steatosis N: 147 (52.3%)	P-value
Hypertension	55 (41)	55 (37.41)	0.53
Dyslipidemia	89 (66.41)	74 (50)	0.006
Obesity	83 (62)	13 (8.8)	0.001

**Table 3:** number of patients according to result of HSI score and hepatic steatosis

		Hepatic steatosis index (HSI)	
		<36	>36
**Steatosis**	Diabetics without hepatic steatosis (N=147)	140	7
	Diabetics with hepatic steatosis (N=134)	14	120

## Discussion

Metabolic fatty liver disease is characterized by an abnormal accumulation of fat in hepatocytes. In the literature, its prevalence in type 2 diabetic patients is between 5 and 87% [4]. It is closely associated with the different components of the metabolic syndrome [5]. Hypertriglyceridemia is a risk factor of development of hepatic steatosis. In our study, hepatic steatosis was significantly correlated with obesity, dyslipidemia and hypertriglyceridemia. Although there is a correlation between fatty liver disease and transaminase levels, most patients with type 2 diabetes have normal serum transaminase levels despite higher liver fat content [5]. The increase in transaminases is not systematically related to the severity of liver disease [6-8]. According to the literature, including Mottin's study showed that 68% of diabetic patients with advanced steatosis have normal levels of transaminases [8,9]. In our patients, abnormal transaminases were objectified in more than 6% of cases. Patients with type 2 diabetes have a higher risk of developing NASH; a death related to fatty liver complications [10,11]. Adams found a twice as high risk of all-cause mortality in patients with T2DM [11]. Another study by Zoppini found similar results with three to five times higher deaths, mainly due to NAFLD [11]. These results suggest that screening for NAFLD should be considered in T2DM. The development of non-invasive serum markers of hepatic damage has facilitated the detection of patients at risk and the follow-up of patients with fatty liver [9]. Imaging may require the precise diagnosis of fatty liver disease, but cannot predict the stage of liver disease. A large cohort has shown a high prevalence of fatty liver disease in type 2 diabetic patients using non-invasive scores [10-12]. Hamaguchi used abdominal ultrasound to assess NAFLD, with a specificity of 100% and a sensitivity of 92% compared to the results of liver biopsy [13,14]. However, conventional imaging is too expensive for mass screening. The hepatic steatosis index is a simple non-invasive tool and a validated screening for NAFLD, with performance characterized by an AUROC of 0.81 (95% confidence interval 0.80-0.836) [15]. HSI score < 30 excludes hepatic steatosis. An HSI score > 36 confirms this with a sensitivity of 93% [15-18]. In our study, the sensitivity of HSI was 89.55%.

## Conclusion

In our study, fatty liver disease was common in patients with type 2 diabetes with normal transaminases. Thus, hepatic steatosis should be screened for in type 2 diabetic patients regardless of their level of transaminases. The non-invasive HSI score is a simple, inexpensive screening for fatty liver, offering the possibility of early diagnosis and prevention of complications related to this condition, it should be part of liver monitoring in patients with type 2 diabetes.

### What is known about this topic

- Metabolic steatopathy is a frequent and often asymptomatic in patients with type 2 diabetes;
- The HSI score is an easy and inexpensive tool offering mass screening for hepatic steatosis.

### What this study adds

- The study reveals that HSI score may be used to facilitate mass screening for hepatic steatosis.
